# Neurocysticercosis: A Review

**DOI:** 10.1100/2012/159821

**Published:** 2012-01-04

**Authors:** Oscar H. Del Brutto

**Affiliations:** ^1^Department of Neurological Sciences, Hospital—Clinica Kennedy, Guayaquil, Ecuador; ^2^Air Center 3542, P.O. Box 522970, Miami, Fl 33152-2970, USA

## Abstract

Neuroysticercosis is the most common helminthic infection of the nervous system, and a leading cause of acquired epilepsy worldwide. The disease occurs when humans become intermediate hosts of *Taenia solium* by ingesting its eggs from contaminated food or, most often, directly from a taenia carrier by the fecal-to-oral route. Cysticerci may be located in brain parenchyma, subarachnoid space, ventricular system, or spinal cord, causing pathological changes that are responsible for the pleomorphism of neurocysticercosis. Seizures are the most common clinical manifestation, but many patients present with focal deficits, intracranial hypertension, or cognitive decline. Accurate diagnosis of neurocysticercosis is possible after interpretation of clinical data together with findings of neuroimaging studies and results of immunological tests. The introduction of cysticidal drugs have changed the prognosis of most patients with neurocysticercosis. These drugs have shown to reduce the burden of infection in the brain and to improve the clinical course of the disease in most patients. Further efforts should be directed to eradicate the disease through the implementation of control programs against all the interrelated steps in the life cycle of *T. solium*, including human carriers of the adult tapeworm, infected pigs, and eggs in the environment.

## 1. Introduction

**Figure 1:**
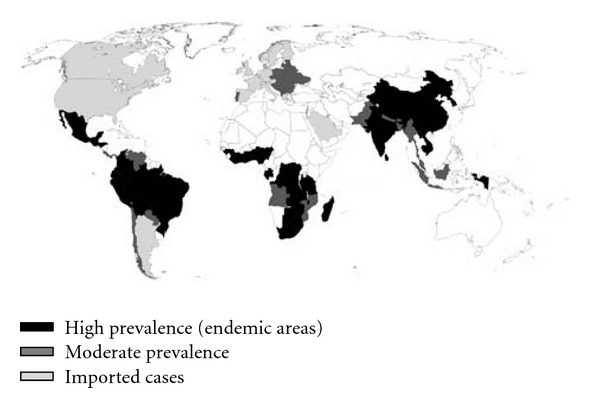
World map showing countries where cysticercosis is endemic.

First recognized as a disease of pork in the ancient Greece, neurocysticercosis is now considered the most common helminthic disease of the central nervous system in humans (Figure 1). The disease is endemic in most of the developing world, where all the conditions favoring the transmission of this parasitosis, including warm climate, severe poverty, and illiteracy are combined. Indeed, population-based studies carried out in rural villages of endemic countries have shown that neurocysticercosis is the main reason for the excess fraction of epilepsy seen in these areas, when compared to the prevalence of epilepsy in developed countries [1–3]. The disease is also a health problem in urban centers of developing countries, where neurocysticercosis is a major cause of admissions to neurological hospitals [4]. Together with the growing number of immigrants from endemic areas, there has been a recent increase in the number of patients with neurocysticercosis in the developed world. Almost 90% of neurocysticercosis patients diagnosed in the US and Europe are Latin American immigrants [5–7]. However, neurocysticercosis has also been recognized in persons with no history of travel to endemic areas, most of whom get infected through a household contact harbouring the adult *Taenia solium* in the intestine [8]. The unpredictable nature of the immunological reaction of the host against cysticerci as well as the pleomorphic lesions that parasites induce in the central nervous system make neurocysticercosis an intriguing disease. During the past years, introduction of modern diagnostic techniques as well as development of cysticidal drugs provoked a considerable interest in neurocysticercosis. Here, we will review the most important aspects of this parasitic disease, with emphasis on its pathogenesis, and on recent advances of diagnosis and therapy.

## 2. Etiopathogenesis

**Figure 2:**
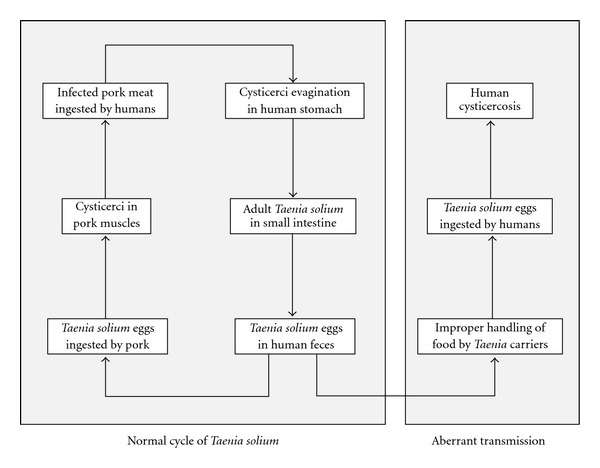
Life cycle of *Taenia solium* showing the normal cycle of transmission, when humans act as definitive hosts and pigs as intermediate hosts, and the aberrant cycle of transmission, when humans become intermediate hosts, developing cysticercosis.

The complex life cycle of *Taenia solium* involves two hosts. Humans are the only definitive hosts for the adult tapeworm, whereas both pigs and humans may act as intermediate hosts for the larval form called cysticercus. In the normal cycle of transmission, the adult *T. solium* inhabits the small intestine of humans, where it is attached to the intestinal wall by its potent suckers and hooks. Gravid proglottids are detached from the distal end of the worm and are passed with the feces, liberating thousands of fertile eggs to the environment. In places with deficient disposal of human feces, pork is fed with human feces containing *T. solium* eggs. Once in the intestinal tract of the pork, the eggs lose their coats and liberate oncospheres which cross the intestinal wall and enter the bloodstream, from where they are carried to the tissues and evolve into cysticercus. Human consumption of improperly cooked infected pork meat results in release of cysticerci in the small intestine, where, by the action of digestive enzymes, their scolices evaginate and attach to the intestinal wall. After the scolex is attached, the proglottids begin to multiply and will become mature approximately four months after infection [9]. Humans can also be intermediate hosts of *T. solium* after ingesting its eggs. Under these circumstances, human cysticercosis develops. Humans acquire cysticercosis from ingestion of food contaminated with *T. solium* eggs or by the fecal-oral route in individuals harboring the adult parasite in the intestine (Figure 2). Recent epidemiological studies, showing clustering of patients with cysticercosis around taeniasic individuals, have changed previous concepts crediting the environment as the main source of human contamination with *T. solium* eggs. Human cysticercosis should be considered as a disease mostly transmitted from person to person, and the role of infected pork is to perpetuate the infection [10].

Cysticerci consist of two main parts, the vesicular wall and the scolex [11]. After entering the central nervous system, cysticerci are in a vesicular (viable) stage in which the parasites have a transparent membrane, a clear vesicular fluid, and a normal invaginated scolex. Cysticerci may remain viable for years or, as the result of the host's immunological attack, enter in a process of degeneration that ends with their transformation into calcifications. The first stage of involution of cysticerci is the colloidal stage, in which the vesicular fluid becomes turbid, and the scolex shows signs of hyaline degeneration. Thereafter, the wall of the cyst thickens and the scolex is transformed into mineralized granules; this stage, in which the cysticercus is not longer viable, is called the granular stage. Finally, the parasite remanents appear as a mineralized nodule (calcified stage) [12].

Vesicular cysticerci elicit little inflammatory reaction in the surrounding tissue. In contrast, colloidal cysticerci are often surrounded by a collagen capsule and by a mononuclear inflammatory reaction that includes the parasite itself. The surrounding brain parenchyma shows astrocytic gliosis, microglial proliferation, edema, neuronal degenerative changes, and perivascular cuffing of lymphocytes. When parasites enter into the granular and calcified stages, the edema subsides but the astrocytic changes in the vicinity of the lesions may become more intense, and epithelioid cells appear and coalesce to form multinucleated giant cells [13]. Meningeal cysticerci usually elicit a severe inflammatory reaction in the subarachnoid space with formation of an exudate composed of collagen fibers, lymphocytes, multinucleated giant cells, eosinophils, and hyalinized parasitic membranes leading to abnormal thickening of the leptomeninges. This inflammation may be disseminated inducing damage in the optic chiasm and cranial nerves arising from the brainstem, as well as in small penetrating arteries arising from the circle of Willis. The latter may cause occlusion of the lumen of the vessel with the subsequent development of a cerebral infarction [14]. The foramina of Luschka and Magendie may also be occluded by the thickened leptomeninges and parasitic membranes with the subsequent development of obstructive hydrocephalus. Ventricular cysticerci may also elicit an inflammatory reaction if they are attached to the choroid plexus or to the ventricular wall. The disrupted ependymal lining may protrude toward the ventricular cavities blocking CSF transit, particularly when the site of protrusion is at or near the foramina of Monro or the cerebral aqueduct [13].

Some cysticercal antigens stimulate the production of specific antibodies that form the basis for the immunological diagnosis of cysticercosis, while others (particularly antigen B) play a role in the evasion of the immune surveillance against cysticerci [15]. In addition, it has been suggested the occurrence of cellular immune dysfunction in patients with neurocysticercosis, resulting from increased subpopulations of CD8 T-lymphocytes, impaired proliferation of lymphocytes, and abnormal concentration of cytokines. It has been hypothesized that this depressed cellular immunity may be responsible for the reported association of neurocysticercosis with conditions resulting from immunodeficiency states, and with the development of gliomas [16]; in such cases, it has been hypothesized that the intense glial proliferation around the parasites, along with the suppression of the cellular immune responses may cause inhibition of the immunological surveillance against cancer, leading to malignant transformation of astrocytes [17].

## 3. Clinical Manifestations

The clinical pleomorphism of neurocysticercosis is mainly related to individual differences in the number and location of the lesions within the CNS and to variations in the severity of disease activity. Seizures are the most common clinical manifestation of neurocysticercosis and may represent the primary or sole manifestation of the disease in almost 70% of patients [18]. Neurocysticercosis is a leading cause of acquired epilepsy in the developing world and, as previously noted, is partly responsible for the increased prevalence of epilepsy seen in developing countries [1–3]. Seizures are more frequently observed in patients with parenchymal neurocysticercosis than in those with subarachnoid or ventricular disease [19]. Epileptogenesis in patients with calcified neurocysticercosis has been a subject of debate [20]. While calcifications have been considered inert lesions, recent data suggest that calcified cysticerci are not clinically inactive nor pathologically inert lesions, as they may cause recurrent seizures when parasitic antigens trapped in the calcium matrix are exposed to the host immune system due to a process of calcification remodeling [21].

Focal neurological signs that vary according to the size, number and location of the parasites have been described in up to 20% patients with neurocysticercosis. Pyramidal tract signs predominate, but sensory deficits, language disturbances, involuntary movements, parkinsonian rigidity, and signs of brainstem dysfunction, may occur in some patients. These manifestations usually follow a subacute or chronic course resembling that of a brain tumor and are most often seen in patients with large subarachnoid cysts compressing the brain parenchyma [22]. Stroke syndromes have also been described in about 3% of patients with neurocysticercosis; these are most often related to cerebral infarctions located in the posterior limb of the internal capsule, the corona radiata, or the brainstem [14]. Some patients with neurocysticercosis develop intracranial hypertension associated or not with seizures or focal neurological signs. The most common cause of this syndrome is hydrocephalus, which may be either related to cysticercotic arachnoiditis, granular ependymitis, or ventricular cysts [22]. Intracranial hypertension also occurs in patients with cysticercotic encephalitis, a severe form of neurocysticercosis that occurs as the result of a massive cysticerci infection of the brain parenchyma inducing a severe immune response from the host. This condition is more frequent among children and young women and is characterized by clouding of consciousness, seizures, diminution of visual acuity, headache, vomiting, and papilledema [23].

Some other patients with neurocysticercosis may present psychiatric manifestations ranging from poor performance on neuropsychological testing to a severe dementia [24]. Before the advent of CT, some of these patients were admitted to psychiatric hospitals for several years until the correct diagnosis was done at autopsy [25]. Patients with cysticerci located in the sellar region present with ophthalmologic and endocrinologic disturbances [26]. Spinal arachnoiditis is characterized by root pain and weakness of subacute onset, and cysts in the spinal cord parenchyma usually present with motor and sensory deficits that vary according to the level of the lesion [27]. Intraocular subretinal cysticerci produce a progressive decrease of visual acuity or visual field defects; ocular cysts may induce vitritis, uveitis, and endophthalmitis [28]. Massive cysticercal infection of striated muscles may produce generalized weakness associated with progressive muscle enlargement [29].

## 4. Diagnosis

**Figure 3:**
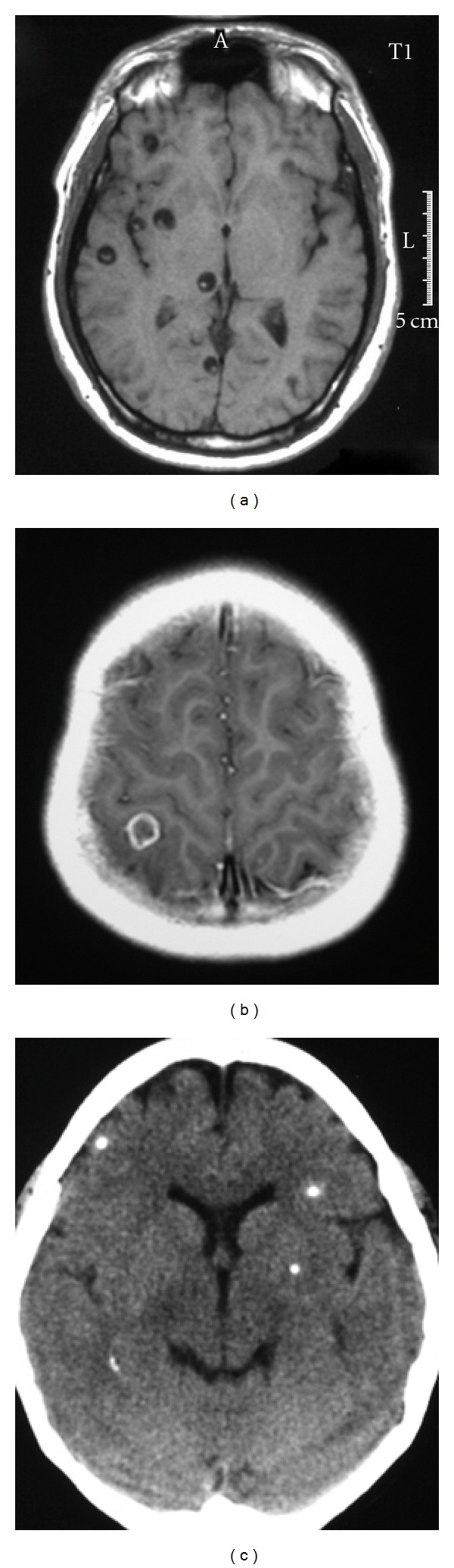
Imaging findings in patients with parenchymal brain cysticercosis, including: viable cysts showing the scolex (a), colloidal cyst appearing as a ring-enhancing lesion (b), and calcifications (c).

The advent of modern neuroimaging tests drastically changed our diagnostic accuracy for neurocysticercosis (Figure 3). CT and MRI provide objective evidence on the number and topography of lesions and their stage of involution [30, 31]. Vesicular cysticerci appear on CT and MRI as small and rounded cysts that are well demarcated from the surrounding brain parenchyma. There is no edema and no contrast enhancement. Many of these lesions have in their interior an eccentric hyperdense nodule representing the scolex, giving them a pathognomonic “hole-with-dot” appearance. Colloidal and granular cysticerci appear as ill-defined lesions surrounded by edema; most of them show a ring or a nodular pattern of enhancement after contrast medium administration. This pattern correspond is commonly referred as to “cysticercus granuloma” [32]. A particular neuroimaging pattern is that observed in patients with cysticercotic encephalitis. CT and MRI show diffuse brain edema, collapse of the ventricular system without midline shift, and multiple small ring-like or nodular enhancing lesions disseminated within the brain parenchyma [23]. Calcified cysticerci normally appear on CT as small hyperdense nodules without perilesional edema or abnormal enhancement after contrast medium administration.

In patients with subarachnoid neurocysticercosis, the most common neuroimaging finding is hydrocephalus related to inflammatory occlusion of Luschka and Magendie foramina. The basal fibrous arachnoiditis that is responsible for the development of hydrocephalus is seen as focal or diffuse areas of abnormal leptomeningeal enhancement. Cystic lesions located within CSF cisterns usually have a multilobulated appearance, displace neighboring structures, and behave as mass occupying lesions [22]. MRA is a valuable noninvasive imaging method to demonstrate segmental narrowing or occlusion of intracranial arteries in patients with subarachnoid neurocysticercosis [14]. Ventricular cysticerci appear on CT as hypodense lesions that distort the ventricular system causing asymmetric hydrocephalus. Since many ventricular cysts are isodense with CSF, they only can be inferred on the basis of distortion on the shape of the ventricular cavities [33]. In contrast, most ventricular cysts are readily visualized on MRI because the signal properties of the cystic fluid or the scolex differ from those of the CSF [34]. Cyst mobility within the ventricular cavities in response to movements of the head, the “ventricular migration sign,” facilitates the diagnosis of ventricular cysticercosis in some cases. Intramedullary cysticerci appear on MRI as rounded lesions that may have an eccentric hyperintense nodule representing the scolex [30]. The periphery of the cyst usually show abnormal enhancement after contrast medium administration. The spinal cord is seen enlarged and, if the scolex is not identified, it may be difficult to differentiate neurocysticercosis from spinal tumors. Leptomeningeal cysts may be mobile within the spinal subarachnoid space and may change their position during the exam according to movements of the patient in the exploration table.

Demonstration that antibodies to species-specific antigens of *T. solium* can be detected by enzyme-linked immunoelectrotransfer blot (EITB) assay stimulated investigators to develop highly purified antigens of cysticercus to be used in a reliable immune diagnostic test for cysticercosis [37]. The main weakness of this test is that it may be false-negative in up to 50% of patients with a single cerebral cyst or in those with calcifications alone [32]. Another weakness is that the test may be positive in patients who had been exposed to the adult parasite without developing cysticercosis [31]. Detection of circulating parasitic antigens using monoclonal antibodies has a poor sensitivity as a screening tool for the diagnosis of neurocysticercosis; however, antigen detection may be of value to monitor the response to cysticidal therapy [38]. The frequency of positive stool exams for *T. solium* eggs among patients with neurocysticercosis has varied from one series to another and seems to be related to the severity of infection [39, 40]. Specific coproantigen detection by ELISA and PCR will improve the screening for *T. solium* carriers among healthy individuals from endemic areas [38].

Despite the abovementioned advances in neuroimaging and immune diagnostic tests, the diagnosis of neurocysticercosis is a challenge in many patients. Clinical manifestations are nonspecific, neuroimaging findings are most often not pathognomonic, and serologic tests are faced with problems related to relatively poor specificity and sensitivity. A set of diagnostic criteria based on the objective evaluation of clinical, radiological, immunological, and epidemiological data has been proposed to provide the physicians with elements to diagnose patients with suspected neurocysticercosis [35]. This set includes four categories of criteria—absolute, major, minor, and epidemiologic—stratified according to their individual diagnostic strengths. Absolute criteria allow unequivocal diagnosis of neurocysticercosis, major criteria strongly suggest the diagnosis but cannot be used alone to confirm the disease, minor criteria are frequent but nonspecific manifestations of the disease, and epidemiologic criteria refer to circumstantial evidence favoring the diagnosis of cysticercosis. Interpretation of these criteria results in two categories of diagnostic certainty—definitive and probable—according to the likelihood that neurocysticercosis is present in a given patient (Table 1).

## 5. Treatment

A single therapeutic approach is not expected to be useful in every patient with neurocysticercosis. Characterization of the disease in terms of viability of cysts, degree of the host's immune response to the parasite, and location and number of lesions is important for rational therapy [41]. Therapy usually include a combination of symptomatic and cysticidal drugs. Surgery has also a role in the management of some patients [42].

The introduction and subsequent widespread use of two potent cysticidal drugs (praziquantel and albendazole) have drastically changed the prognosis of most patients with neurocysticercosis [43]. The initial regimen of praziquantel at doses of 50 mg/kg/day (given every 8 hours) for 15 days was arbitrarily chosen [44]. It was then suggested that if cysticerci are exposed to high concentrations of the drug maintained for up to 6 hours by giving 3 individual doses of 25 to 30 mg/kg at two-hour intervals, this might be sufficient to destroy the parasites. While preliminary results with this new regimen were encouraging [45], it seems that the single-day course of praziquantel works better for patients with a single parenchymal brain cyst, and that the 15-day trial should be used for those with more than one cysts [46]. Albendazole, the other cysticidal drug, was initially administered at doses of 15 mg/kg/day during one month [47]. Further studies showed that the length of therapy could be shortened to one week without lessening the efficacy of the drug [48], and even to three days if the patient has a single brain cyst [49]. Albendazole has been superior to praziquantel in trials comparing the efficacy of these drugs [50, 51]. Another advantage of albendazole is that it also destroys subarachnoid and ventricular cysts [52]. In some of these cases, particularly in patients with large subarachnoid cysts, higher doses (up to 30 mg/kg/day) or more prolonged, or even repeated, courses of albendazole may be needed [22, 53, 54].

Due to the benign nature of some forms of neurocysticercosis, the use of cysticidal drugs has been questioned, leading to confusion and incorrect decisions in the management of many patients. It has been claimed that cysticidal drugs only destroy the cysts without modifying the clinical course of the disease [55]. Nevertheless, more recent studies have shown that cysticidal drugs also produce clinical improvement in most patients. In a placebo-controlled trial, albendazole was effective for therapy of viable parenchymal brain cysticerci [56]. Other controlled trials showed that the prognosis of patients with colloidal parenchymal brain cysts is better after therapy than when the disease is left untreated [36, 57, 58]. A recent meta-analysis of randomized trials evaluated the effect of cysticidal drugs on neuroimaging and clinical outcomes of patients with neurocysticercosis [59]. According to that meta-analysis, published evidence indicates that cysticidal drug therapy results in better resolution of both colloidal and vesicular cysticerci, in a lower risk of seizure recurrence in patients with colloidal cysticerci, and in a reduction in the rate of generalized seizures in patients with vesicular cysticerci.

It must be remembered that some forms of neurocysticercosis should not be treated with cysticidal drugs [41]. These drugs may exacerbate the syndrome of intracranial hypertension observed in patients with cysticercotic encephalitis. In patients with both hydrocephalus and parenchymal brain cysts, cysticidal drugs may be used only after a ventricular shunt has been placed to avoid further increases of the intracranial pressure as a result of therapy. Cysticidal drugs must be used with caution in patients with giant subarachnoid cysticerci because the inflammatory reaction developed by the host in response to the acute destruction of the parasite may occlude leptomeningeal vessels surrounding the cyst; concomitant steroid administration is mandatory to avoid the hazard of a cerebral infarct. In patients with ventricular cysts, the use of cysticidal drugs should be individualized. While albendazole successfully destroys many ventricular cysts, the inflammatory reaction may cause acute hydrocephalus if the cysts are located within the fourth ventricle or near the foraminae of Monro. Finally, patients with calcifications alone should not receive cysticidal drugs since these lesions represent already dead parasites (Garcia et al., [41]).

The administration of a single first-line antiepileptic drug usually results in control of seizures in patients with neurocysticercosis-related epilepsy. There is some evidence that patients with viable intracranial cysts should first be treated with cysticidal drugs to achieve an adequate control of seizures with antiepileptic drugs [18, 56]. The optimal length of antiepileptic drug therapy in patients with neurocysticercosis has not been settled. A prospective study showed that up to 50% of patients with parenchymal brain cysticerci successfully treated with cysticidal drugs had relapses after withdrawal of antiepileptic drugs [60]. Prognostic factors associated with seizure recurrence include the development of parenchymal brain calcifications and the presence of both recurrent seizures and multiple brain cysts before the institution of therapy. In patients with single-enhancing lesions (colloidal cysts), the development of brain calcification after therapy is also the main determinant for seizure relapse after withdrawal of antiepileptic drugs; in such cases, long-term antiepileptic treatment may be needed [32].

## 6. Control Measures

Neurocysticercosis is common in areas where conditions favoring the transmission of *T. solium* are found, including deficient disposal of human feces, low levels of education, slaughtering of pigs without veterinary control, and presence of free roaming pigs around households [19]. This parasitic disease is potentially eradicable. To be effective, however, eradication programs must be directed to all the targets for control, particularly human carriers of the adult tapeworm, infected pigs, and eggs in the environment [61, 62]. Since these targets represent interrelated steps in the life cycle of *T. solium*, inadequate coverage of one of them may result in a rebound in the prevalence of taeniosis/cysticercosis after the program has been completed.

## Figures and Tables

**Table 1:** Diagnostic criteria and degrees of diagnostic certainty for neurocysticercosis (Modified from: [35]).

Diagnostic criteria	
Absolute	
(i) Histologic demonstration of the parasite from biopsy of a brain or spinal cord lesion.	
(ii) Evidence of cystic lesions showing the scolex on neuroimaging studies.	
(iii) Direct visualization of subretinal parasites by fundoscopic examination.	
(iv) Spontaneous resolution of small single enhancing lesions.	

Major	
(i) Evidence of lesions highly suggestive of neurocysticercosis on neuroimaging studies.	
(ii) Positive serum immunoblot for the detection of anticysticercal antibodies.	
(iii) Resolution of intracranial cystic lesions after therapy with albendazole or praziquantel.	

Minor	
(i) Evidence of lesions suggestive of neurocysticercosis on neuroimaging studies.	
(ii) Presence of clinical manifestations suggestive of neurocysticercosis.	
(iii) Positive CSF ELISA for detection of anticysticercal antibodies or cysticercal antigens.	
(iv) Evidence of cysticercosis outside the central nervous system.	

Epidemiologic	
(i) Individuals coming from or living in an area where cysticercosis is endemic.	
(ii) History of frequent travel to disease-endemic areas.	
(iii) Evidence of household a contact with *T. solium* infection.	

Degrees of diagnostic certainty	

Definitive	
(i) Presence of one absolute criterion.	
(ii) Presence of two major plus one minor or one epidemiologic criteria.	

Probable	
(i) Presence of one major plus two minor criteria.	
(ii) Presence of one major plus one minor and one epidemiologic criteria.	
(iii) Presence of three minor plus one epidemiologic criteria.	
